# Selection of an adjuvant for seasonal influenza vaccine in elderly people: modelling immunogenicity from a randomized trial

**DOI:** 10.1186/1471-2334-13-348

**Published:** 2013-07-26

**Authors:** Hans C Rümke, Jan Hendrik Richardus, Lars Rombo, Karlis Pauksens, Georg Plaßmann, Christelle Durand, Jeanne-Marie Devaster, Walthère Dewé, Lidia Oostvogels

**Affiliations:** 1 Medical Director, Vaxinostics, University Vaccine Center, Rotterdam, The Netherlands; 2 Senior Investigator, Department of Infectious Disease Control, Municipal Public Health Service (GGD) Rotterdam-Rijnmond, Rotterdam, The Netherlands; 3 Clinical research centre Sormland, Uppsala University, Uppsala, Sweden; 4 Department of Medical Sciences, Section of Infectious Diseases, Uppsala University, Akademiska Sjukhuset, Uppsala, Sweden; 5 Unterfrintroper Hausarztzentrum, Unterstraße 75, 45359, Essen, Germany; 6 Vaccine Value & Health Science, GlaxoSmithKline Vaccines, Wavre, Belgium; 7 Vaccine Discovery and Development, GlaxoSmithKline Vaccines, Wavre, Belgium

**Keywords:** Influenza vaccination, Elderly, Adjuvant system, Dose comparison, Immunogenicity, Reactogenicity, Safety

## Abstract

**Background:**

Improved influenza vaccines are needed to reduce influenza-associated complications in older adults. The aim of this study was to identify the optimal formulation of adjuvanted seasonal influenza vaccine for use in elderly people.

**Methods:**

This observer-blind, randomized study assessed the optimal formulation of adjuvanted seasonal influenza vaccine based on immunogenicity and safety in participants aged ≥65 years. Participants were randomized (~200 per group) to receive one dose of non-adjuvanted vaccine or one of eight formulations of vaccine formulated with a squalene and tocopherol oil-in-water emulsion-based Adjuvant System (AS03_C_, AS03_B_ or AS03_A_, with 2.97, 5.93 and 11.86 mg tocopherol, respectively) together with the immunostimulant monophosphoryl lipid A (MPL, doses of 0, 25 or 50 mg). Hemagglutination-inhibition (HI) antibody responses and T-cell responses were assessed on Day 0 and 21 days post-vaccination. The ratio of HI-based geometric mean titers in adjuvanted versus non-adjuvanted vaccine groups were calculated and the lower limit of the 90% confidence interval was transformed into a desirability index (a value between 0 and 1) in an experimental domain for each vaccine strain, and plotted in relation to the AS03 and MPL dose combination in the formulation. This model was used to assess the optimal formulation based on HI antibody titers. Reactogenicity and safety were also assessed. The immunogenicity and safety analyses were used to evaluate the optimal formulation of adjuvanted vaccine.

**Results:**

In the HI antibody-based model, an AS03 dose–response was evident; responses against the A/H1N1 and A/H3N2 strains were higher for all adjuvanted formulations versus non-adjuvanted vaccine, and for the AS03_A_-MPL25, AS03_B_-MPL25 and AS03_B_-MPL50 formulations against the B strain. Modelling using more stringent criteria (*post hoc*) showed a clear dose-range effect for the AS03 component against all strains, whereas MPL showed a limited effect. Higher T-cell responses for adjuvanted versus non-adjuvanted vaccine were observed for all except two formulations (AS03_C_ and AS03_B_-MPL25). Reactogenicity increased with increasing AS03 dosage, and with MPL. No safety concerns were raised.

**Conclusions:**

Five formulations containing AS03_A_ or AS03_B_ were identified as potential candidates to improve immune responses to influenza vaccination; AS03_B_ without MPL showed the best balance between improved immunogenicity and acceptable reactogenicity.

**Trial registration:**

This trial is registered at ClinicalTrials.gov, NCT00540592

## Background

A significant number of the seasonal influenza-associated hospitalizations in the United States occur among older adults, and influenza-associated mortality also disproportionately affects this age group [1,2]. Inactivated trivalent influenza vaccines have been available for over 60 years, but are reported to be less effective in older adults than in younger adults. This is generally attributed to the weaker immune responses to vaccination in older individuals due to an age-related decline in immune function [3-5]. This immunosenescence may also underlie the increased severity of influenza-related complications observed in older people [5]. Nonetheless, influenza vaccination remains a cost-saving medical intervention in older adults that results in significant reductions in hospitalizations and deaths due to complications of influenza illness [6-8].

Given the influenza-related morbidity and mortality rates in older people despite widespread vaccination, there is clearly a need to develop improved influenza vaccines that are more effective in this vulnerable population. Strategies to enhance vaccine immunogenicity and overcome the limitations of immunosenescence include the use of high antigen doses and the formulation of vaccine with an adjuvant. For several decades, aluminum salt was the only adjuvant approved for human use, but this failed to improve the immunogenicity of influenza vaccines [9]. Recent advances in vaccine technology have led to the incorporation of new adjuvants or Adjuvant Systems into influenza vaccines; these include oil-in-water emulsions containing squalene, a naturally occurring triterpene and a key precursor in hepatic cholesterol biosynthesis, together with non-ionic surfactant emulsifiers. The squalene-based oil-in-water adjuvant MF59™ is reported to improve the immunogenicity of seasonal influenza vaccines compared with non-adjuvanted vaccine [10], and a tocopherol-based oil-in-water Adjuvant System (AS03) is currently licensed for use with avian-origin H5N1 (*Prepandrix™,* a trade mark of the GlaxoSmithKline group of companies*)* and swine-origin A(H1N1)pdm09 (*Pandemrix™,* a trade mark of the GlaxoSmithKline group of companies) pandemic influenza vaccines [11]. *Prepandrix*™ and *Pandemrix*™ have been shown to improve immunogenicity compared with non-adjuvanted vaccine in healthy adults, and are highly immunogenic in elderly populations [12-16]. More recently, an AS03-adjuvanted, low antigen dose (15 μg/strain) seasonal influenza vaccine was compared with a licensed seasonal influenza vaccine (45 μg antigen: 15 μg/vaccine), and immunogenicity with the low dose adjuvanted vaccine was shown to be non-inferior to the ‘normal dose’ non-adjuvanted vaccine in people aged ≥60 years [17]. Therefore, it is possible that adjuvantation may enhance immunogenicity beyond that provided by existing vaccines in older people.

Oil-in-water emulsions can also accommodate additional adjuvant-active molecules, such as monophosphoryl lipid A (MPL). MPL is a nontoxic derivative of lipopolysaccharide from *Salmonella minnesota* and a powerful stimulator of the immune system that is known to act as a toll-like receptor agonist [18]. MPL has been used as a component of a hepatitis B vaccine (approximately 44 900–224 500 doses administered since licensure up to 1^st^ February 2012) and a human papillomavirus vaccine (approximately 9.6–28.95 million doses up to 17^th^ November 2011) [18]. These MPL-containing vaccines were well-tolerated with local reactogenicity symptoms generally mild to moderate in intensity and of a short duration, and no safety concerns were raised in clinical trials [19,20]. Based on the enhanced immunogenicity reported for both MPL and AS03, we were interested to know whether the addition of MPL would further enhance the immune system response to AS03.

The objective of this study was to identify an optimal formulation of adjuvanted influenza vaccine for use in older adults. Eight different formulations of seasonal influenza vaccine, adjuvanted with AS03 oil-in-water emulsion with or without MPL, were evaluated in adults aged 65 years and older with regard to immunogenicity, safety and reactogenicity. The control vaccine was a commercially available non-adjuvanted seasonal influenza vaccine.

## Methods

### Study objectives

The aim of this observer-blind, randomized study was to select the optimal doses of both AS03 and MPL to be used in an adjuvanted influenza vaccine for use in older people. The immunogenicity, reactogenicity and safety of different formulations of adjuvanted influenza vaccine administered to participants aged ≥65 years were evaluated and compared with non-adjuvanted influenza vaccine. The primary outcome was hemagglutination-inhibition (HI) based immunogenicity against all three vaccine strains at 21 days after vaccination. A contour plot model was used to assess the optimal adjuvanted formulation based on HI antibody titers. Secondary objectives included assessment of cell-mediated responses, safety and reactogenicity.

### Vaccines

The split virus influenza vaccine (*Fluarix*™, a trade mark of the GlaxoSmithKline group of companies) was formulated with eight different adjuvants, or given as plain vaccine (hereafter, non-adjuvanted). All vaccines contained 15 μg hemagglutinin antigen (HA) from three influenza strains (45 μg HA in total): A/Solomon Islands/03/2006 (IVR-145) (H1N1 strain), A/Wisconsin/67/2005 (NYMC-X161B) (H3N2 strain) and B/Malaysia/2506/2004 (B strain). The compositions of the AS03 Adjuvant Systems used for the eight adjuvanted formulations are shown in Table 1. Each of the adjuvanted influenza vaccines were presented as a vial containing the antigens and a syringe containing the adjuvant. The contents of the pre-filled syringe, containing the adjuvant, was injected into the vial that contained the antigens, and after mixing, the entire contents were withdrawn into the syringe to give one 0.7 mL dose of reconstituted, adjuvanted influenza vaccine. The non-adjuvanted vaccine was presented as a pre-filled syringe (0.5 mL). All vaccines were prepared and administered intramuscularly by unblinded personnel who took no further part in the study procedures; participants and personnel involved in assessment of safety, data processing and analysis were blind to vaccine allocation.

**Table 1:** Composition of the adjuvanted influenza vaccine formulations

**Formulation**	**α -tocopherol (mg)**	**MPL (μg)**
AS03_C_	2.97	0
AS03_C_-MPL25	2.97	25
AS03_C_-MPL50	2.97	50
AS03_B_	5.93	0
AS03_B_-MPL25	5.93	25
AS03_B_-MPL50	5.93	50
AS03_A_	11.86	0
AS03_A_-MPL25	11.86	25

All formulations of the vaccine contained AS03, which is a squalene and α-tocopherol oil-in-water emulsion-based Adjuvant System. MPL is 3-O-desacyl-4’- monophosphoryl lipid A.

### Participants and study design

The study was conducted in Germany (21 centers: 11 general practices, 2 centers, 2 not specified), the Netherlands (2 centers: 1 clinical research center, 1 Municipal Health Service center), Sweden (3 centers: 2 hospital clinics, 1 clinical research center) and the United Kingdom (5 clinical research centers). The first participant was enrolled in October 2007, and the final study visit was in June 2008.

Volunteers were eligible to participate in the study if they were aged 65 years or older, were free of an acute aggravation of health status as established by clinical evaluation and provided written informed consent. Besides inclusion of participants from the target age group of ≥65 years, a control group of young adults aged 18–40 years old was included as a reference group to compare the immune response and reactogenicity of the adjuvanted vaccines.

Eligible participants aged 65 years or older were randomized to receive one dose of non-adjuvanted vaccine or one of eight different formulations of adjuvanted influenza vaccine (Table 1); the group aged 18–40 years received non-adjuvanted vaccine. Vaccine allocation was performed by the sponsor using a central internet randomization system. The nine groups aged ≥65 years and the 18–40 years group were randomized 1:1:1:1:1:1:1:1:1:1. A randomization algorithm used a blocking scheme to ensure a balance between the vaccines, and stratified the study groups (aged ≥65 years) by age (65–74 years [65%] and ≥75 years [35%]). The algorithm incorporated a minimization procedure to account for center. The study was observer-blind for participants aged ≥65 years and open-label for participants aged 18–40 years.

The protocol and study documents were approved by the appropriate Independent Ethics Committees or Institutional Review Boards in each country (Ethikkommission bei der Sächsischen Landesärztekammer, Oxfordshire Research Ethics Committee B, Regionala Etikprövningsnämnden I Uppsala and Centrale Commissie Mensgebonden Onderzoek) (Additional file 1). The study was conducted in accordance with the Declaration of Helsinki. GlaxoSmithKline Biologicals SA sponsored the study and was involved in all stages of the study conduct, including analysis of the data. All authors had full access to the data and were involved in the analysis of the data and preparation of the manuscript. The trial is registered at ClinicalTrials.gov, NCT00540592. A summary of the protocol may be found at: http://www.gsk-clinicalstudyregister.com/ (study ID 110847).

### Immunogenicity

Blood samples were collected for immunological testing, before (Day 0) and 21 days after (Day 21) vaccination. All assays were performed in a GlaxoSmithKline Vaccines central laboratory or in a validated laboratory designated by GlaxoSmithKline Vaccines, using standardized, validated procedures with validated controls.

HI assays were performed on serum samples as described previously using chicken red blood cells [21]. Antibody responses were described as geometric mean titer (GMT; anti-log of the mean of the log-10 transformed titers), seroprotection rate (SPR; proportion of participants with post-vaccination titer ≥1:40), seroconversion rate (SCR; proportion of participants with titer <1:10 at baseline with post-vaccination titer of ≥1:40, or pre-vaccination titer of ≥1:10 and ≥4-fold increase post-vaccination), and seroconversion factor (SCF; geometric mean of the within subject ratios of reciprocal HI antibody titers for post-vaccination versus pre-vaccination).

Cell mediated immune (CMI) responses were based on T cell responses, as described previously [22,23]. Briefly, blood samples were collected in heparinized tubes and kept at ambient temperature (15–25°C), until peripheral blood mononuclear cells (PBMCs) were isolated (within 24 h of blood sampling) and frozen at −80°C. Immunological assays were performed later in test runs with PBMC/T cells in one run with adequate control cells. PBMCs were re-stimulated *ex vivo* by incubation with influenza antigen in the presence of co-stimulatory antibodies to CD28 and CD49d, and Brefeldin A. Cells were subsequently harvested, stained for surface markers (CD4) and fixed. Fixed cells were then permeabilized and stained with antibodies specific for interferon-gamma (IFN-γ), interleukin-2 (IL-2), tumor necrosis factor-alpha (TNF-α) or CD40L, and analyzed by cytofluorometry. Results were expressed as the frequencies of influenza-specific CD4 T cells producing at least two response markers.

### Reactogenicity and safety

Participants used diary cards to record the occurrence and intensity of injection site solicited adverse events (ecchymosis, pain, redness and swelling) and systemic solicited adverse events (arthralgia, fatigue, headache, myalgia, nausea, shivering and fever) during the first 7 days after vaccination. The diameters of any injection site ecchymosis, redness or swelling were recorded, with grade 3 defined as >100 mm. Daily body temperature was also recorded; grade 3 fever was defined as body temperature ≥39.0–40.0°C, and grade 4 fever as body temperature >40.0°C. The intensities of other adverse events were recorded according to a standard 0 to 3 grade scale: “absent”, “easily tolerated”, “interferes with normal activity” or “prevents normal activity”. Data were also collected on the occurrence and intensity of any spontaneous unsolicited signs. Data on serious adverse events (SAEs) were collected prospectively from 21 days to 180 days after vaccination.

### Statistical analyses

The target sample size was 2000 participants, including 200 participants in the 18–40 years stratum, 1170 participants in the 65–74 years stratum, and 630 participants in the ≥75 years stratum. The expected precision of the relative immune response estimation was presented for 190 evaluable participants in each group (assuming that about 5% of subjects would not be evaluable for the immunogenicity endpoints). This was based on computations of the lower limits of the 90% confidence interval (CI) of the geometric mean titer (GMT) ratio (AS03 adjuvanted vaccine over non-adjuvanted vaccine in the ≥65 year group), according to various expected values for the GMT ratio estimate and various standard deviations at the log10-scale (one-sided alpha = 5%).

Reactogenicity and safety was assessed in the total vaccinated cohort including all participants who were vaccinated. HI antibody responses were evaluated in the per protocol immunogenicity cohort including vaccinated participants who complied with the study protocol and for whom a serological sample was available at a given time point. CMI responses were assessed in a subset of 500 participants from pre-specified study centers; the analysis was performed on the per protocol cell mediated immunogenicity cohort including vaccinated participants in the CMI subset who complied with the study protocol and for whom a serological sample was available at a given time point.

HI-based GMT, SCR, SCF, and SPR were calculated with 95% CIs as defined by regulatory criteria [24,25]. GMT ratios for each formulation versus non-adjuvanted vaccine were calculated with two-sided 90% CIs. The frequencies of immune response marker-positive CD4 T cells producing at least two immune response markers (all doubles) were calculated as medians with interquartile ranges for Days 0 and 21. The reactogenicity and safety endpoints were the percentages of participants reporting an event with 95% CIs.

#### Immunogenicity model

A multi-criteria decision-making approach was used to identify which vaccine combination of AS03 and MPL would have the most desirable immune response 21 days after vaccination compared with the non-adjuvanted vaccine in participants aged ≥65 years. HI antibody titers were used to produce a contour plot of overall desirability to identify the region of the experimental domain with the highest desirability, *i.e.* the region containing the formulations demonstrating the best HI immunogenicity for the three vaccine strains. Within the domain of variation of both AS03 and MPL, the least square means of the log titers and the standard errors were estimated using a multiple regression model. The arithmetic mean of the log transformed HI titers for the non-adjuvanted vaccine was calculated as well as its standard error. Then the 90% CI lower limits (LL) of the ratio for each AS03 and MPL combination versus the non-adjuvanted vaccine were transformed into a desirability index between 0 and 1 using a sigmoidal Derringer function. Each LL was transformed to a value between 0 and 1, where 0 indicated an undesirable relative immune response, and 1 a highly desirable relative immune response. In the prospective analysis, an LL equal to 1.0 was transformed into a desirability equal to 0.5. A *post hoc* analysis was also performed using more stringent criteria, where an LL equal to 1.5 was transformed in desirability equal to 0.5 (applied only for A strains).

An overall desirability index was obtained by taking a weighted geometric mean of the three desirability indexes associated to the three vaccine strains. A contour plot of the desirability indexes in the experimental domain was then built for each strain and for all three strains using a color spectrum from red denoting the most undesirable index to purple denoting the most desirable index. Based on the color contour plots combining the three strains, formulations were then ranked according to the overall desirability index. The GMTs, SCRs, SCFs, and SPRs of the formulations ranked most desirable were further evaluated to assess the adequacy of the expectation.

## Results

**Figure 1:**
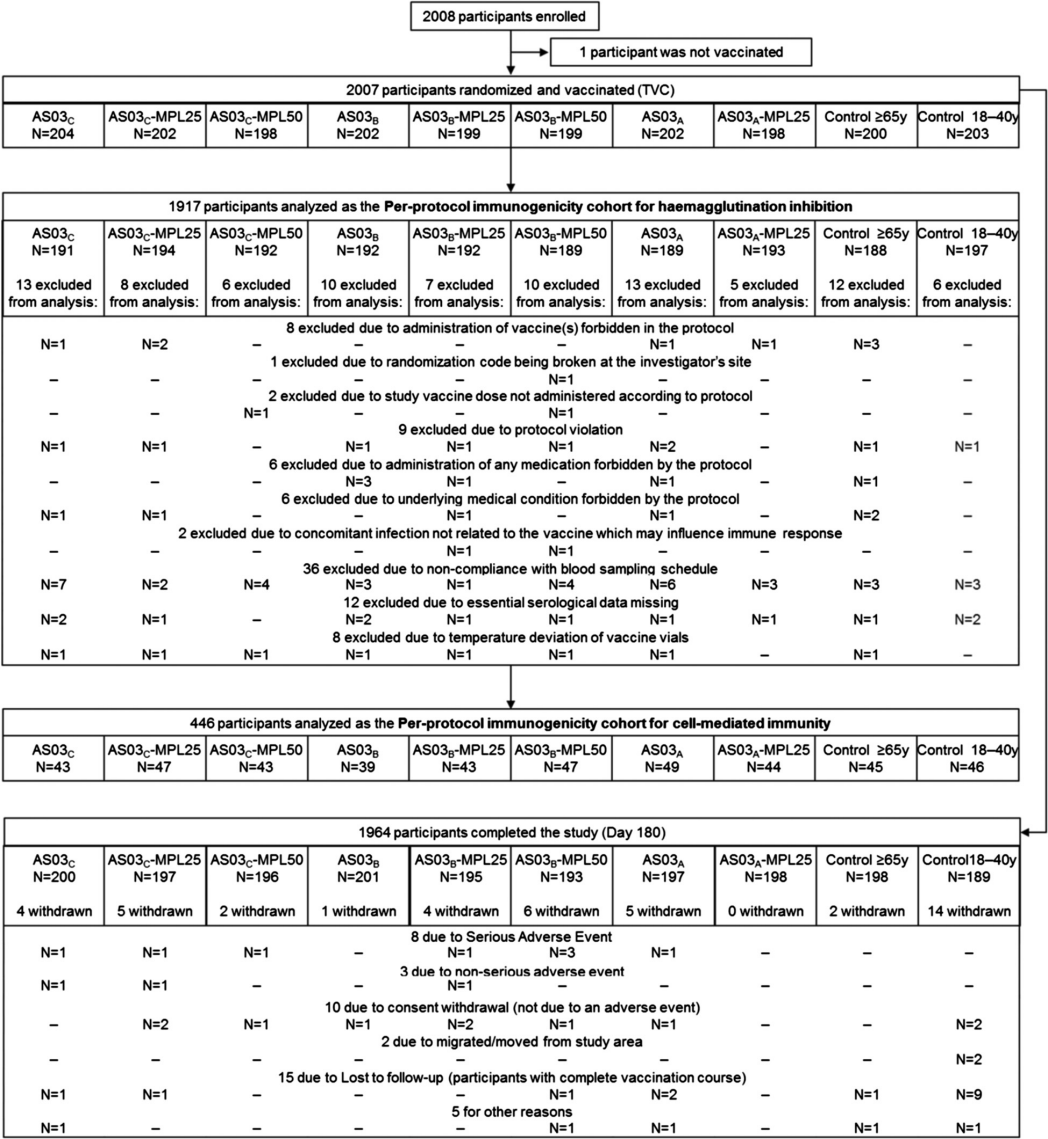
**Participant flow.***Footnote:* TVC, total vaccinated cohort; all participants received inactivated trivalent influenza vaccine; control ≥65 years and control 18–40 years received non-adjuvanted vaccine; eight groups aged ≥65 years received vaccine formulated with an adjuvant. AS03 is a squalene and α-tocopherol oil-in-water emulsion-based Adjuvant System (tocopherol content: A, 11.86 mg; B, 5.93 mg; C, 2.97 mg); MPL is 3-O-desacyl-4’- monophosphoryl lipid A (MPL content: 25, 25 μg; 50, 50 μg).

A total of 2008 participants were enrolled, including 203 aged 18–40 years, 1177 aged 65–74 years, and 627 aged ≥75 years, of which 1997 participants completed the study visit at Day 21 (Figure 1). Ninety participants were eliminated from the per protocol immunogenicity cohort for HI for non-compliance with the protocol (Figure 1). The mean age of the participants at the time of vaccination was 72.7 ± 5.66 years (median 72.00 years) for the participants aged ≥65 years and 27.4 ± 6.25 years (median 27.00 years) for participants aged 18–40 years. Excluding the 18–40 years group, the mean age at vaccination in the individual vaccine groups ranged from 72.4–73.0 years in the total vaccinated cohort (Table 2). The majority of participants (>97%) were White-Caucasian. The ≥65 years vaccine groups were balanced for demographic characteristics.

**Table 2:** Demographic characteristics in the total vaccinated cohort

	**AS03**_**C**_	**AS03**_**C**_**-MPL25**	**AS03**_**C**_**-MPL50**	**AS03**_**B**_	**AS03**_**B**_**-MPL25**	**AS03**_**B**_**-MPL50**	**AS03**_**A**_	**AS03**_**A**_**-MPL25**	**Non-adjuvanted ≥65 years**	**Non-adjuvanted 18–40 years**
	N=204	N=202	N=198	N=202	N=199	N=199	N=202	N=198	N=200	N=203
**Age at vaccination (years)**										
Mean±SD	72.7±5.75	72.9±6.09	72.6±5.67	72.6±5.16	72.5±5.66	73.0±6.03	72.4±5.62	72.7±5.56	72.7±5.43	27.4±6.25
Median (min–max)	72.0	71.0	72.0	72.0	72.0	72.0	71.0	72.0	72.0	27.0
	(63–91)	(64–91)	(65–94)	(65–87)	(65–91)	(62–95)	(65–90)	(65–88)	(65–89)	(18–40)
**Gender, n (%)**										
Female	95 (46.6)	100 (49.5)	96 (48.5)	109 (54.0)	89 (44.7)	95 (47.7)	105 (52.0)	102 (51.5)	90 (45.0)	103 (50.7)
Male	109 (54.3)	102 (50.5)	102 (51.5)	93 (46.0)	110 (55.3)	104 (52.3)	97 (48.0)	96 (48.5)	110 (55.0)	100 (49.3)
**Geographic Ancestry, n (%)**										
African/African American	1 (0.5)	0	2 (1.0)	0	0	1 (0.5)	0	1 (0.5)	0	2 (1.0)
Asian–Central/South	1 (0.5)	0	0	0	0	0	0	0	0	0
Asian–South East	1 (0.5)	1 (0.5)	2 (1.0)	0	0	1 (0.5)	2 (1.0)	0	0	0
White–Arabic/North African	2 (1.0)	1 (0.5)	1 (0.5)	1 (0.5)	1 (0.5)	1 (0.5)	0	3 (1.5)	0	2 (1.0)
White–Caucasian/European	199 (97.5)	199 (98.5)	193 (97.5)	201 (99.5)	197 (99.0)	195 (98.0)	198 (98.0)	194 (98.0)	200 (100.0)	197 (97.0)
Other	0	1 (0.5)	0	0	1 (0.5)	1 (0.5)	2 (1.0)	0	0	2 (1.0)

TVC, total vaccinated cohort; All participants received inactivated trivalent influenza vaccine, non-adjuvanted (≥65 years and 18–40 years) or formulated with an adjuvant. AS03 is a squalene and α-tocopherol oil-in-water emulsion-based Adjuvant System, with tocopherol content 11.86 mg (A), 5.93 mg (B), or 2.97 mg (C); MPL is 3-O-desacyl-4’- monophosphoryl lipid A: 25 μg (MPL-25) or 50 μg (MPL-50).

### Humoral immunogenicity

**Figure 2:**
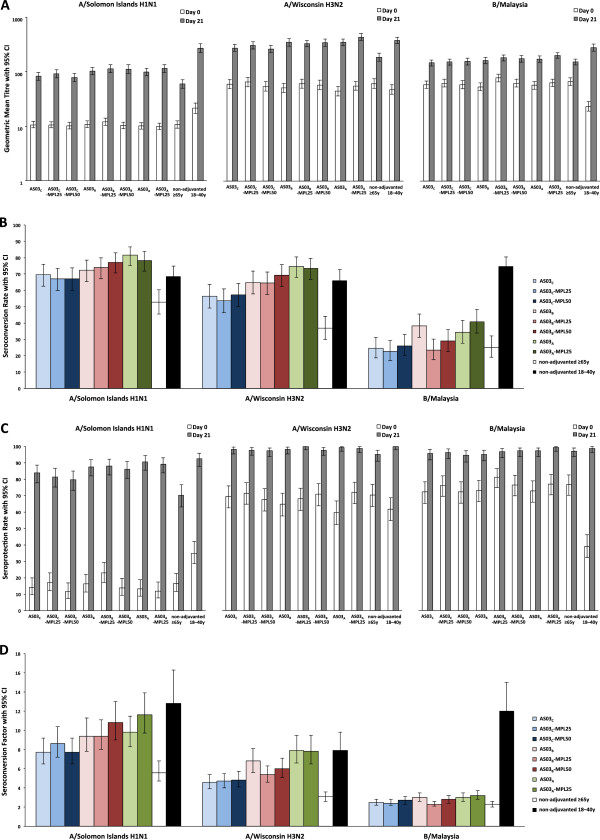
**HI antibody responses in the per protocol immunogenicity cohort : Geometric mean titers (A); Seroconversion rates (B); Seroprotection rates (C); Seroconversion factor (D).***Footnote:* HI, Hemagglutination-inhibition; TVC, total vaccinated cohort; All participants received inactivated trivalent influenza vaccine, non-adjuvanted (≥65 years and 18–40 years) or formulated with an adjuvant. AS03 is a squalene and α-tocopherol oil-in-water emulsion-based Adjuvant System, with tocopherol content 11.86 mg (A), 5.93 mg (B), or 2.97 mg (C); MPL is 3-O-desacyl-4’- monophosphoryl lipid A: 25 μg (MPL-25) or 50 μg (MPL-50).

The HI antibody responses to each of influenza vaccine strains in each study group are shown in Figure 2 (data are detailed in Additional file 2). In recipients of non-adjuvanted vaccine, stronger immune responses were seen in the younger group than in the ≥65 years group; Day 21 GMTs for ≥65 years versus 18–40 years were 59.6 *vs.* 271.6 for the A/Solomon Islands H1N1 strain, 186.7 *vs.* 380.2 for the A/Wisconsin H3N2 strain, and 153.0 *vs.* 281.9 for the B/Malaysia strain.

#### Immunogenicity modelling

**Figure 3:**
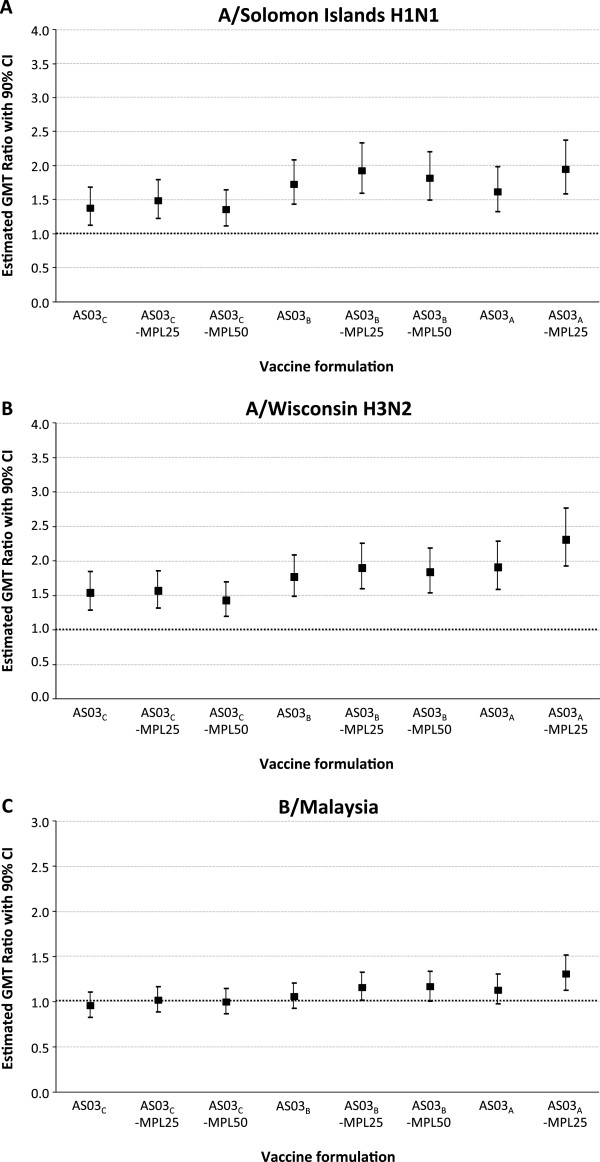
**HI GMT ratio for adjuvanted versus non-adjuvanted formulation in the per protocol immunogenicity cohort against A/H1N1 (A), A/H3N2 (B), and influenza B strain (C).***Footnote:* HI, hemagglutination-inhibition; All participants received inactivated trivalent influenza vaccine, non-adjuvanted (≥65 years and 18–40 years) or formulated with an adjuvant. AS03 is a squalene and α-tocopherol oil-in-water emulsion-based Adjuvant System, with tocopherol content 11.86 mg (A), 5.93 mg (B), or 2.97 mg (C); MPL is 3-O-desacyl-4’- monophosphoryl lipid A: 25 μg (MPL-25) or 50 μg (MPL-50).

**Figure 4:**
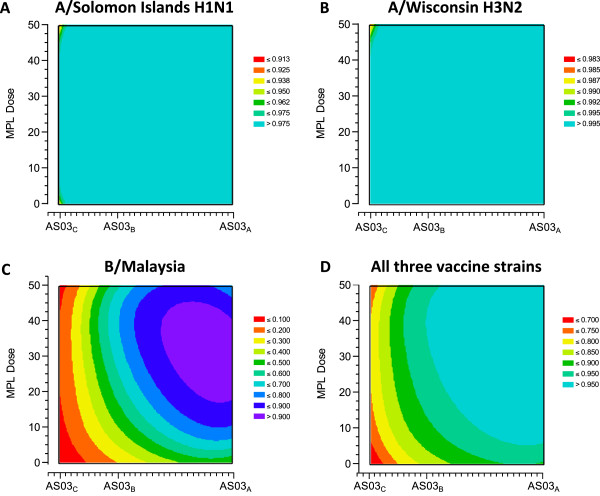
**Contour plots of HI antibody responses in the per protocol immunogenicity cohort against A/H1N1 (A), A/H3N2 (B), influenza B (C), and all vaccine strains (D).***Footnote:* HI, hemagglutination-inhibition; GMT ratios for each adjuvanted formulation versus non-adjuvanted vaccine calculated with two-sided 90% Confidence Interval (CI); the lower limits (LL) of CI were transformed into a desirability index between 0 and 1 using a sigmoidal Derringer function; each LL was transformed to a value between 0 and 1, where 0 indicated an undesirable relative immune response, and 1 a highly desirable relative immune response. LL equal to 1.0 was transformed into a desirability equal to 0.5. The colors range from red (least desirable) to purple (most desirable).

The GMT ratios for adjuvanted versus non-adjuvanted vaccine are shown in Figure 3. Contour plots are shown in Figure 4. A high desirability index was observed in the contour plots for the A/Solomon Islands H1N1 and A/Wisconsin H3N2 strains at all AS03 and MPL doses compared with the non-adjuvanted ≥65 year group in all the different formulation groups. All 90% CIs had a LL >1 which was higher than the predefined highest desirability score. In contrast, the contour plot for the B/Malaysia strain showed that the highest desirability was obtained with formulations containing higher concentrations of AS03 (AS03_B_ and AS03_A_) together with MPL. This was associated with the higher geometric mean ratio with LL >1.0 for the AS03_A_-MPL25, AS03_B_-MPL25 and AS03_B_-MPL50 formulations. These two observations lead to the conclusion that only the B strain contributed to the overall ranking.

**Figure 5:**
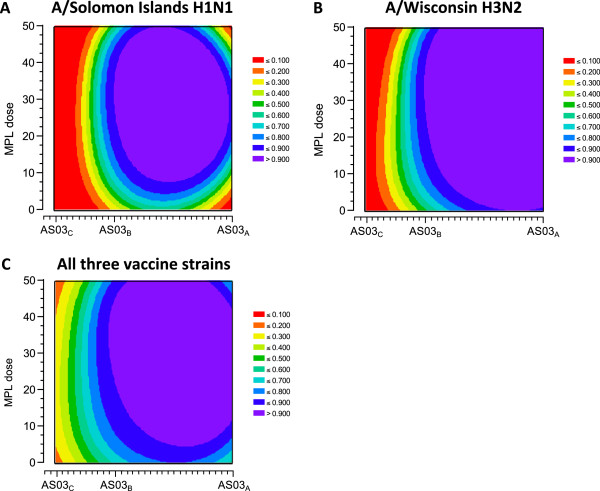
**Contour plots (*post-hoc*) of HI antibody responses in the per protocol immunogenicity cohort against A/H1N1 (A), A/H3N2 (B), and all three vaccine strains (C).***Footnote:* HI, hemagglutination-inhibition; GMT ratios for each adjuvanted formulation versus non-adjuvanted vaccine calculated with two-sided 90% Confidence Interval (CI); the lower limits (LL) of CI were transformed into a desirability index between 0 and 1 using a sigmoidal Derringer function; each LL was transformed to a value between 0 and 1, where 0 indicated an undesirable relative immune response, and 1 a highly desirable relative immune response. For A/H1N1 and A/H3N2, an LL equal to 1.5 was transformed in desirability equal to 0.5. The colors range from red (least desirable) to purple (most desirable).

More discriminative contour plots were generated based on *post hoc* analyses performed with more stringent conditions for the A/Solomon Islands H1N1 and A/Wisconsin H3N2 strains (a desirability of 0.5 was associated with a LL=1.5) (Figure 5). A clear dose-range effect was observed for the AS03 component, whereas MPL showed limited or no effect. This was reflected in the *post hoc* plot for all three vaccine strains (Figure 5).

Overall, a dose-range effect of the oil-in-water component was found, with the weakest immunogenicity exhibited by the AS03_C_ formulations. When all three strains were considered together, the desirability plots indicated that the AS03_A_-MPL25 formulation had the highest score (Figure 5C).

### Cell-mediated immunogenicity

**Figure 6:**
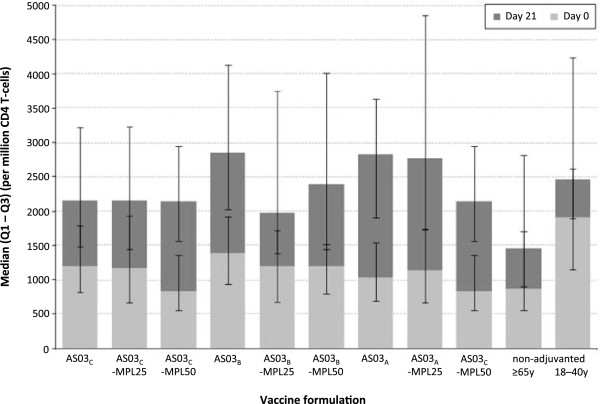
**Cell mediated immunogenicity in the per protocol cell mediated immunogenicity cohort.***Footnote:* Results for each time point are indicated by median values with first and third quartiles. All participants received inactivated trivalent influenza vaccine, non-adjuvanted (≥65 years and 18–40 years) or formulated with an adjuvant. AS03 is a squalene and α-tocopherol oil-in-water emulsion-based Adjuvant System, with tocopherol content 11.86 mg (A), 5.93 mg (B), or 2.97 mg (C); MPL is 3-O-desacyl-4’- monophosphoryl lipid A: 25 μg (MPL-25) or 50 μg (MPL-50).

The frequencies of influenza-specific CD4 T cells producing at least two immune response markers in each group, at Day 0 and Day 21 following stimulation by the three pooled strains are shown in Figure 6. Vaccination with all except two of the formulations (AS03_C_ and AS03_B_-MPL25) resulted in the observation of higher CD4 T cell responses compared with the non-adjuvanted ≥65 year group following *ex vivo* stimulation with all three pooled strains. For three of the formulations (AS03_A,_ AS03_B_ and AS03_A_-MPL25), the pooled influenza strain-specific CD4 T cell responses were also found to be higher than the non-adjuvanted 18–40 years group.

### Safety and reactogenicity

During the 21-day follow-up period, 19 participants reported 25 SAEs, ranging from 0–2.0% per study group (Table 3). The outcome of one SAE (pancreatic tumor in the AS03_B_-MPL25 group diagnosed 17 days after the first dose of vaccine), which led to withdrawal from the study, was subsequently fatal, but judged by the investigator not to be related to vaccination. Two further participants (1 each in the AS03_B_ and AS03_B_-MPL50 groups) withdrew from the study due to SAEs (atrial fibrillation/ischemic stroke and myocardial infarction) that were not related to vaccination. One SAE (chest pain) was considered by the investigator to be possibly causally related to vaccination, since the chest pain started on the day of vaccination (AS03_B_-MPL50). This event resolved within 9 days. Between Days 21 and 179, 112 participants reported 134 SAEs, ranging from 1.5–9.5% per study group. There were 4 fatal SAEs during this time, none of which were considered to be related to vaccination (1 participant in each of the AS03_C_ and AS03_C_-MPL25 groups and 2 participants in the AS03_B_-MPL50 group). Two additional participants withdrew from the study due to a SAE (worsening of gout and abdominal pain). One SAE (chronic myelomonocytic leukemia) was considered by the investigator to be possibly related to vaccination, as the participant felt that there was an association between the time of vaccination (non-adjuvanted ≥65 year group) and the development of symptoms. The onset of the SAE was Day 69, and the SAE had not recovered/resolved by the end of the study.

**Table 3:** Participants reporting at least one serious adverse event from Day 0 to Day 179 in the total vaccinated cohort

	**AS03**_**C**_	**AS03**_**C**_**-MPL25**	**AS03**_**C**_**-MPL50**	**AS03**_**B**_	**AS03**_**B**_**-MPL25**	**AS03**_**B**_**-MPL50**	**AS03**_**A**_	**AS03**_**A**_**-MPL25**	**Non-adjuvanted ≥65 y**	**Non-adjuvanted 18–40 y**
	N=204	N=202	N=198	N=202	N=199	N=199	N=202	N=198	N=200	N=203
Day 0–21	0 (0.0%)	2 (1.0%)	2 (1.0%)	3 (1.5%)	2 (1.0%)	4 (2.0%)	2 (1.0%)	4 (2.0%)	0 (0.0%)	0 (0.0%)
Day 21–179	18 (8.8%)	8 (4.0%)	16 (8.1%)	9 (4.5%)	9 (4.5%)	19 (9.5%)	5 (2.5%)	10 (5.1%)	15 (7.5%)	3 (1.5%)

All participants received inactivated trivalent influenza vaccine, non-adjuvanted (≥65 years and 18–40 years) or formulated with an adjuvant. AS03 is a squalene and α-tocopherol oil-in-water emulsion-based Adjuvant System, with tocopherol content 11.86 mg (A), 5.93 mg (B), or 2.97 mg (C); MPL is 3-O-desacyl-4’- monophosphoryl lipid A: 25 μg (MPL-25) or 50 μg (MPL-50).

**Figure 7:**
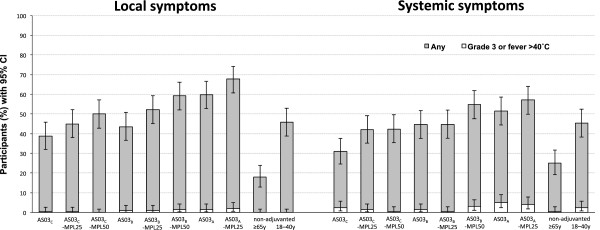
**Participants reporting solicited and unsolicited symptoms during the 7-day post-vaccination period in the total vaccinated cohort.***Footnote:* CI, confidence interval; All participants received inactivated trivalent influenza vaccine, non-adjuvanted (≥65 years and 18–40 years) or formulated with an adjuvant. AS03 is a squalene and α-tocopherol oil-in-water emulsion-based Adjuvant System, with tocopherol content 11.86 mg (A), 5.93 mg (B), or 2.97 mg (C); MPL is 3-O-desacyl-4’- monophosphoryl lipid A: 25 μg (MPL-25) or 50 μg (MPL-50).

The reactogenicity profile during the 7 days following vaccination is shown in Figure 7. The percentage of participants reporting at least one local symptom (solicited or unsolicited) ranged from 38.7% to 67.7% in the adjuvanted vaccine groups, 18.0% reported a symptom in the non-adjuvanted ≥65 year group, and 45.8% in the non-adjuvanted 18–40 years group. For systemic symptoms, between 30.9% and 57.1% of participants reported a symptom in the adjuvanted vaccine groups, 25.0% in the non-adjuvanted ≥65 year group, and 45.3% in the non-adjuvanted 18–40 years group. The incidence of grade 3 events and fever >40°C was between 0 and 5.0% for all groups. A dose-range effect may be observed on the reactogenicity of the adjuvanted formulations, with a trend towards increasing incidence of symptoms with increasing AS03 content and with the presence of MPL. This was also reflected in the solicited symptom profiles.

Pain was the most common solicited local symptom (Additional file 3). The maximum number of reports (60.4%) was in the AS03_A_-MPL25 group, with 16.0% in the non-adjuvanted ≥65 year group, and 46.3% in the non-adjuvanted 18–40 years group. The three most commonly reported general solicited symptoms (Additional file 4) were myalgia (maximum: 33.3% in the AS03_A_-MPL25 group *vs.* 9.5% in the ≥65 year group and 19.9% in the 18–40 years group), fatigue (maximum: 31.8% in the AS03_B_-MPL50 group *vs.* 12.0% in the non-adjuvanted ≥65 year group and 20.4% in the 18–40 years group) and headache (maximum: 27.8% in the AS03_A_-MPL25 group *vs.* 11.5% in the non-adjuvanted ≥65 year group and 22.4% in the 18–40 years group).

## Discussion

This randomized study assessed eight different formulations of influenza vaccine, adjuvanted with AS03 (squalene and tocopherol oil-in-water emulsion) with or without MPL, and a contour plot model was used to identify an optimal formulation for vaccination of older adults based on immunogenicity. Age-related declines in innate, and adaptive humoral and cell-mediated immunity, is thought to impair the ability to resist influenza infection and response to vaccination [26,27], therefore, the rationale for formulating seasonal influenza vaccine with the adjuvant was to enhance immunogenicity and potentially improve protection of existing vaccines.

HI antibody responses with non-adjuvanted vaccine in the younger (18–40 years) control group were consistent with responses reported by randomized immunogenicity trials with this vaccine (*Fluarix*™), and responses in older (≥65 years) people to non-adjuvanted control vaccine were relatively low; seroprotection rates (proportion with titer ≥1:40) were 92.4–100% in the 18–40 year old group compared with 70.2–97.3% in the older group. In older participants, HI antibody responses to all adjuvanted formulations appeared slightly higher than responses to non-adjuvanted vaccine; however, because 200 participants per group did not provide sufficient power to compare the groups, a statistical model based on HI GMTs was used to objectively rank the different formulations. In the model, the lower limit of the 90% CI for the GMT ratio for each adjuvanted formulation versus non-adjuvanted was used to assign a ‘desirability index’ for each formulation, which involved transforming the CI to a value between 0 and 1, where 0 was undesirable and 1 was most desirable. Contour plots were used to characterize the desirability of each formulation based on the immunogenicity against each vaccine strain, and all three strains.

The contour plots showed that the effect of adjuvantation on the humoral response to vaccination was strain-dependent. For HI antibody responses against the A/Solomon Islands H1N1 and A/Wisconsin H3N2 strains, all AS03 formulations had the highest desirability, but for the B/Malaysia strain, an increase in desirability was observed when the AS03 dose approached a maximum level. The model also indicated that whereas inclusion of MPL appeared to increase desirability based on HI responses against the B/Malaysia strain, no clear effect was shown for the A/Solomon Islands H1N1 and the A/Wisconsin H3N2 strains.

The unexpected observations for the influenza A strains lead to a lack of discrimination within the initial plots, so we performed *post hoc* analyses applying more stringent criteria to further evaluate potential differences between the formulations. In the *post hoc* model, a higher AS03 dose, but not MPL, increased the desirability based on HI responses against both of the influenza A strains. Overall for the three vaccine strains, the dosage of MPL had a limited effect in discriminating the formulations in terms of HI response despite the fact that the AS03_A_-MPL25 formulation had the highest score. The contour plots showed that AS03 dosage had a clear effect with relatively high AS03 content formulations needed to achieve an optimal HI antibody response. Increased immune responses with increasing AS03 dosage was further confirmed by the HI immunogenicity results, although the sample size in each group did not allow definite statistical conclusions.

In addition to the provision of CD4 T cell help for B cell differentiation, both CD4 effector and memory T cells appear to have multifaceted roles in the protective responses to influenza infection [23,28]. CD4+ T-helper cells are polarized into T helper 1 or T helper 2 cells, and T helper 1 cells predominantly secrete IFN-γ, which activates macrophages and facilitates clearance of intracellular pathogens [29-32]. Impairment of T cell responses due to aging is therefore considered to be an important factor that limits the responses to vaccination, and it has been proposed that correlates of vaccine-mediated protection against influenza in older adults should include measures of cell-mediated responses [5,33]. In our study, adjuvanted vaccine also enhanced CD4 T cell-mediated responses to all three vaccine strains compared with non-adjuvanted vaccine in elderly people, with the most marked impact on responses to the pooled strains was observed for the AS03_A,_ AS03_B_ and AS03_A_-MPL25 formulations. This indicates that the presence of MPL does not appear, in this setting, to be essential to achieve maximal enhancement of CD4 T cell responses to influenza antigens.

Oil-in-water adjuvants have been shown to enhance the response to vaccination by triggering chemokine production resulting in recruitment of immunocompetent cells to the injection site, cell maturation into a dendritic phenotype, the stimulation of antigen uptake into these cells, and facilitation of subsequent migration to the lymph nodes [22,34-36]. The adjuvant activity of AS03 is highly dependent on the presence of the immunostimulant α-tocopherol (an isoform of vitamin E) [37], which distinguishes AS03 from other oil-in-water emulsion-based adjuvants. The oil-in-water adjuvant MF59™ contains polysorbate 80, sorbitan trioleate, trisodium citrate dehydrate, citric acid monohydrate, and has been shown to enhance immune responses to seasonal influenza vaccine compared with non-adjuvanted vaccine [10,38]. An MF59™-adjuvanted influenza vaccine (Fluad™) is licensed and widely used, and has been shown to significantly reduce hospitalization for influenza and pneumonia compared with non-adjuvanted vaccine in elderly people [39]. However, there have been no prospective, randomized clinical trials of vaccine efficacy of this formulation in people aged ≥65 years [10].

Seasonal influenza vaccines are licensed based on demonstrating the induction of HI antibody titers above a defined threshold; a HI antibody titer of 1:40 is generally accepted as corresponding to a 50% reduction in the risk of influenza, which is based on a challenge study conducted forty years ago [40]. However, although humoral immune responses measured by HI may be a good correlate of protection in younger adults, it has been suggested that HI titers may not be an adequate surrogate for protection against influenza in older adults. Indeed, vaccine efficacy data from field trials are considered to provide a more meaningful measure of the benefits of influenza vaccination than serological data in elderly populations. Based on the results of our Phase II study, AS03_B_ (5.93 mg tocopherol) without MPL was selected for further evaluation as it provided the best balance between the immune response and reactogenicity; AS03-adjuvanted versus non-adjuvanted trivalent influenza vaccine was evaluated in a Phase III study including 43,000 people in 15 countries, representing the largest field study to date to assess influenza vaccine efficacy in people aged ≥65 years (NCT00753272). In addition to vaccine efficacy, the study evaluated clinical outcomes, immunogenicity, reactogenicity and safety [41].

In addition to enhancing immunogenicity, the incorporation of adjuvant components into a vaccine can also have an impact on the safety/reactogenicity profile. We observed that in the 7 days following vaccination, overall reactogenicity was higher for the eight adjuvanted influenza formulations than for non-adjuvanted influenza vaccine in ≥65 year old participants. In general, the incidence of most reactions in the adjuvanted vaccine groups remained within the same range as those induced by non-adjuvanted control vaccine in the young adult group. A dose-range effect was observed, with the highest incidence of reactions associated with the AS03_A_ formulations. The presence of MPL also tended to increase reactogenicity of the influenza vaccine. Nevertheless, the majority of adverse events reported following administration of all eight adjuvanted formulations were mild to moderate in nature, and no clinically observable safety concerns were raised. Ultimately, assuming similar safety profiles, the public acceptability of the reactogenicity profiles for the different formulations in the days immediately following vaccination will depend upon the extent of the clinical benefit. The selection of final candidate formulations must therefore be based on a balance between improvement in immunogenicity and increase in reactogenicity.

Limitations of the current study included that the randomization procedure did not take previous vaccination history into account, or that there may have been a tendency for more healthy participants to be recruited, which cannot be adjusted for in the analyses. Another possible limitation of the study is that a relatively low sample size was used in the testing of cell-mediated immunity. Although it is not a limitation, it should be noted that the GMT ratios used in the primary endpoint was calculated using 90% CIs instead of the more usual 95% CIs. The desirability model was based only on the GMT ratios, and the reactogenicity results were descriptive. In addition, descriptive immunogenicity (SCR, SCF and SPR) was based on HI antibody assays, as this measure is the basis of the established correlate of protection in seasonal influenza; other measures of immunogenicity such as microneutralization assay and single radial haemolysis were not used.

## Conclusions

Five formulations containing AS03_A_ or AS03_B_ with or without MPL were considered to be potential candidates to improve immune responses to seasonal influenza vaccine in older adults. The statistical model used helped rank the different formulations with a reasonable study sample size. For further development, AS03_B_ without MPL was selected as the optimal formulation for use in the older adult population, based on the balance between improved immunogenicity and acceptable reactogenicity.

## Abbreviations

ASO3: Adjuvant system 03; CI: Confidence interval; GMT: Geometric mean titer; GSK: GlaxoSmithKline; HA: Hemagglutinin antigen; HI: Hemagglutination-inhibition; IFN-γ: Interferon-gamma; IL: Interleukin; LL: Lower limit; MPL: 3-O-desacyl-4’-monophosphoryl lipid A; PBMC: Peripheral blood mononuclear cell; PGE2: Prostaglandin E2; SAE: Serious adverse event; SCF: Seroconversion factor; SCR: Seroconversion rate; SPR: Seroprotection rate; TVC: Total vaccinated cohort.

## Competing interests

The authors declare the following competing interests: JHR: received travel grants from GlaxoSmithKline Biologicals SA to attend investigator meetings; received grants from GlaxoSmithKline Biologicals SA to perform vaccine trials. LR: received payment from GlaxoSmithKline Biologicals SA for giving lectures on vaccinations for primary health care; received a travel grant from GlaxoSmithKline Biologicals SA to attend a congress.

KP: received grants from GlaxoSmithKline Biologicals SA to perform clinical trials and has performed clinical trials for Pfizer, Sanofi Pasteur and Eurocine. GP: received grants from GlaxoSmithKline Biologicals SA to perform vaccine trials. CD, JMD, WD and LO are employed by the GlaxoSmithKline group of companies. JMD, WD and LO own stock options for GlaxoSmithKline Biologicals SA.

GSK Biologicals SA was the funding source and was involved in all stages of the study conduct and analysis. GSK Biologicals SA also took in charge all costs associated with the development and the publishing of the present manuscript. The corresponding author had full access to the data and had final responsibility to submit for publication.

All participating institutions received compensation for study involvement.

## Authors’ contributions

J-M Devaster, W Dewé, C Durand, L Oostvogels and HC Rümke participated in the conception, design and planning of the study. J-M Devaster, L Oostvogels, K Pauksens, G Plassmann, JH Richardus, L Rombo and HC Rümke collected and assembled data. J-M Devaster, W Dewé, C Durand, L Oostvogels, K Pauksens and HC Rümke performed and supervised the analysis. J-M Devaster, W Dewé, C Durand, L Oostvogels, K Pauksens, JH Richardus, L Rombo and HC Rümke interpreted data. G Plassmann and JH Richardus provided administrative, technical or logistic support. J-M Devaster acquired funding and contributed to the recruitement of centers and investigators. J-M Devaster and L Oostvogels supervised the study. K Pauksens, G Plassmann, JH Richardus, L Rombo and HC Rümke provided study materials or subjects. C Durand provided statistical expertise. All authors reviewed the manuscript during its development and approved its final draft.

## Pre-publication history

The pre-publication history for this paper can be accessed here:

http://www.biomedcentral.com/1471-2334/13/348/prepub

## Supplementary Material

Additional file 1 Independent Ethics Committees/Institutional Review Boards. Click here for file

Additional file 2 HI antibody responses in the per protocol immunogenicity cohort. Click here for file

Additional file 3 Participants reporting solicited local symptoms during the 7-day post-vaccination period in the total vaccinated cohort. Click here for file

Additional file 4 Participants reporting solicited systemic symptoms during the 7-day post-vaccination period in the total vaccinated cohort. Click here for file
