# Supplementary material for: Selection of an adjuvant for seasonal influenza vaccine in elderly people: modelling immunogenicity from a randomized trial
Source: BMC Infect Dis. 2013 Jul 26;13:348. doi: 10.1186/1471-2334-13-348 (PMC3729430; doi:10.1186/1471-2334-13-348)
Supplement: Additional file 1 — Independent Ethics Committees/Institutional Review Boards. [file 1471-2334-13-348-S1.pdf]

Additional file 1. Independent Ethics Committees/Institutional Review Boards

No. of centres	Ethics review body	Location
3	Ethik-Kommission der Ärztekammer Hamburg	Heinrich-Hertz-Str. 125, 22083 Hamburg, Germany.
4	Landesamt für Gesundheit und Soziales, Geschäftsstelle der Ethik-Kommission des Landes Berlin	Sächsische Str. 28, 10707 Berlin, Germany.
2	Ethik-Kommission der Landesärztekammer Rheinland-Pfalz	Deutschhausplatz 3, 55116 Mainz, Germany.
4	Ethik-Kommission bei der Landesärztekammer Baden-Württemberg	Jahnstraße 40, 70597 Stuttgart, Germany.
2	Ethik-Kommission des Landes Sachsen-Anhalt c/o Landesamt für Verbraucherschutz	Kühnauer Str. 70, 06846 Dessau, Germany.
2	Ethikkommission der Ärztekammer Nordrhein	Tersteegenstraße 9, 40474 Düsseldorf, Germany.
4	Ethikkommission bei der Sächsischen Landesärztekammer	Schützenhöhe 16, 01099 Dresden, Germany.
1	Ethik-Kommission der Bayerischen Landesärztekammer	Mühlbaurstraße 16, 81677 München, Germany.
2	Centrale Commissie Mensgebonden Onderzoek (CCMO)	Parnassusplein 5, 2511 VX Den Haag, The Netherlands.
3	Regionala Etikprövningsnämnden i Uppsala	Box 1964 SE-751 49 Uppsala Sweden.
5	Oxfordshire REC B	2nd Floor Astral House, Chaucer Business Park, Granville Way, Bicester, United Kingdom.
