# Supplementary material for: Selection of an adjuvant for seasonal influenza vaccine in elderly people: modelling immunogenicity from a randomized trial
Source: BMC Infect Dis. 2013 Jul 26;13:348. doi: 10.1186/1471-2334-13-348 (PMC3729430; doi:10.1186/1471-2334-13-348)
Supplement: Additional file 2 — HI antibody responses in the per protocol immunogenicity cohort. [file 1471-2334-13-348-S2.pdf]

Additional file 2. HI antibody responses in the per protocol immunogenicity cohort

		**AS03$_{C}$**	**AS03$_{C}$-MPL25**	**AS03$_{C}$-MPL50**	**AS03$_{B}$**	**AS03$_{B}$-MPL25**	**AS03$_{B}$-MPL50**	**AS03$_{A}$**	**AS03$_{A}$-MPL25**	**Non-adjuvanted**	**Non-adjuvanted 18–40y**
		N=191	N=194	N=192	N=192	N=192		N=189	N=189	N=187	N=197
**A/Solomon Islands**											
GMT, value (95% CI)	D0	10.6<br>(9.3, 12.2)	10.6<br>(9.3, 12.1)	10.2<br>(8.9, 11.7)	10.8<br>(9.4, 12.4)	12 .0<br>(10.3, 14.0)	10.3<br>(9.0, 11.9)	10.1<br>(8.8, 11.5)	9.9<br>(8.6, 11.3)	10.7<br>(9.2, 12.4)	21.3<br>(17.1, 26.5)
	D21	82.3<br>(69.7, 97.1)	91.7<br>(76.9, 109.3)	78.5<br>(65.9, 93.5)	102.2<br>(87.0, 119.9)	113.2<br>(96.0, 133.6)	111.8<br>(93.3, 134.0)	98.2<br>(84.3, 114.3)	114.4<br>(97.1, 134.8)	59.6<br>(49.6, 71.7)	271.6<br>(224.6, 328.4)
SCR, % (95% CI)	D21	69.6<br>(62.6, 76.1)	67.0<br>(59.9, 73.6)	67.2<br>(60.1, 73.8)	72.3<br>(65.3, 78.5)	74.0<br>(67.1, 80.0)	77.2<br>(70.6, 83.0)	81.5<br>(75.2, 86.7)	78.2<br>(71.7, 83.8)	52.9<br>(45.5, 60.3)	68.5<br>(61.5, 74.9)
SPR, % (95% CI)	D0	14.1<br>(9.5, 19.9)	17.0<br>(12.0, 23.1)	11.5<br>(7.3, 16.8)	16.2<br>(11.3, 22.2)	22.9<br>(17.2, 29.5)	13.8<br>(9.2, 19.5)	13.2<br>(8.7, 18.9)	11.9<br>(7.7, 17.3)	16.6<br>(11.6, 22.7)	35.0<br>(28.4, 42.1)
	D21	83.8<br>(77.8, 88.7)	81.4<br>(75.2, 86.7)	79.7<br>(73.3, 85.1)	87.5<br>(82.0, 91.8)	88.0<br>(82.6, 92.3)	86.2<br>(80.5, 90.8)	90.5<br>(85.4, 94.3)	89.1<br>(83.8, 93.1)	70.2<br>(63.1, 76.6)	92.4<br>(87.8, 95.7)
SCF, % (95% CI)	D21	7.7<br>(6.5, 9.2)	8.6<br>(7.2, 10.4)	7.7<br>(6.5, 9.2)	9.4<br>(7.8, 11.3)	9.4<br>(8.0, 11.1)	10.8<br>(9.0, 13.0)	9.8<br>(8.3, 11.5)	11.6<br>(9.7, 13.9)	5.6<br>(4.7, 6.8)	12.8<br>(10.0, 16.3)
**A/Wisconsin**											
GMT, value (95% CI)	D0	59.9<br>(49.5, 72.4)	65.7<br>(53.7, 80.4)	54.6<br>(44.5, 67.0)	51.2<br>(41.9, 62.4)	61.1<br>(50.6, 73.8)	57.8<br>(47.1, 70.9)	44.4<br>(36.5, 54.0)	55.8<br>(46.2, 67.4)	61.0<br>(49.8, 74.7)	47.8<br>(39.2, 58.4)
	D21	276.2<br>(238.8, 319.6)	309.2<br>(264.9, 361.0)	263.3<br>(225.3, 307.7)	350.2<br>(298.0, 411.4)	331.1<br>(289.6, 378.6)	347.5<br>(296.4, 407.3)	351.3<br>(304.8, 404.8)	438.1<br>(379.7, 505.4)	186.7<br>(158.0, 220.7)	380.2<br>(331.6, 435.9)
SCR, % (95% CI)	D21	56.5<br>(49.2, 63.7)	53.6<br>(46.3, 60.8)	57.3<br>(50.0, 64.4)	64.9<br>(57.7, 71.7)	64.6<br>(57.4, 71.3)	69.3<br>(62.2, 75.8)	74.6<br>(67.8, 80.6)	73.6<br>(66.8, 79.6)	36.9<br>(30.0, 44.2)	66.0<br>(58.9, 72.6)
SPR, % (95% CI)	D0	69.6<br>(62.6, 76.1)	71.6<br>(64.8, 77.9)	67.7<br>(60.6, 74.3)	64.9<br>(57.7, 71.7)	68.2<br>(61.1, 74.7)	70.9<br>(63.9, 77.3)	59.8<br>(52.4, 66.8)	72.0<br>(65.1, 78.2)	70.6<br>(63.5, 77.0)	61.9<br>(54.8, 68.7)

		**AS03$_C$**	**AS03$_C$-MPL25**	**AS03$_C$-MPL50**	**AS03$_B$**	**AS03$_B$-MPL25**	**AS03$_B$-MPL50**	**AS03$_A$**	**AS03$_A$-MPL25**	**Non-adjuvanted**	**Non-adjuvanted 18–40y**
	D21	98.4	97.9	97.4	98.4	100	97.9	99.5	99.0	95.2	100
		(95.5, 99.7)	(94.8, 99.4)	(94.0, 99.1)	(95.5, 99.7)	(98.1, 100)	(94.7, 99.4)	(97.1, 100)	(96.3, 99.9)	(91.1, 97.8)	(98.1, 100)
SCF, %	D21	4.6	4.7	4.8	6.8	5.4	6.0	7.9	7.8	3.1	7.9
(95% CI)		(3.9, 5.4)	(4.0, 5.5)	(4.1, 5.7)	(5.6, 8.1)	(4.6, 6.3)	(5.1, 7.1)	(6.6, 9.5)	(6.5, 9.5)	(2.6, 3.6)	(6.4, 9.8)
**B/Malaysia**											
GMT,	D0	58.6	62.6	58.2	53.7	77.8	62.6	57.0	62.9	66.5	23.4
value		(49.8, 68.9)	(53.3, 73.4)	(49.0, 69.1)	(45.7, 63.2)	(66.0, 91.7)	(53.0, 74.0)	(48.2, 67.5)	(53.9, 73.4)	(56.5, 78.3)	(19.1, 28.7)
(95% CI)											
	D21	147.1	152.4	155.7	163.1	181.4	174.6	172.1	202 .0	153.0	281.9
		(129.1, 167.6)	(134.4, 172.7)	(134.8, 179.8)	(142.5, 186.6)	(158.9, 207.1)	(152.0, 200.7)	(149.9, 197.6)	(179.7, 227.1)	(135.2, 173.1)	(241.7, 328.7)
SCR, %	D21	24.6	22.7	26.0	38.2	23.4	29.1	34.4	40.9	25.1	74.6
(95% CI)		(18.7, 31.3)	(17.0, 29.2)	(20.0, 32.9)	(31.3, 45.5)	(17.6, 30.1)	(22.7, 36.1)	(27.6, 41.6)	(33.9, 48.2)	(19.1, 32.0)	(67.9, 80.5)
SPR, %	D0	72.3	76.3	72.4	73.3	81.3	76.7	73.0	77.2	77.0	39.1
(95% CI)		(65.3, 78.5)	(69.7, 82.1)	(65.5, 78.6)	(66.4, 79.4)	(75.0, 86.5)	(70, 82.5)	(66.1, 79.2)	(70.6, 82.9)	(70.3, 82.8)	(32.2, 46.3)
	D21	95.8	96.4	94.8	95.3	96.9	97.4	97.4	99.5	97.3	99.0
		(91.9, 98.2)	(92.7, 98.5)	(90.6, 97.5)	(91.3, 97.8)	(93.3, 98.8)	(93.9, 99.1)	(93.9, 99.1)	(97.1, 100)	(93.9, 99.1)	(96.4, 99.9)
SCF, %	D21	2.5	2.4	2.7	3.0	2.3	2.8	3.0	3.2	2.3	12.0
(95% CI)		(2.2, 2.8)	(2.2, 2.8)	(2.3, 3.1)	(2.6, 3.5)	(2.1, 2.6)	(2.4, 3.2)	(2.6, 3.5)	(2.8, 3.7)	(2.0, 2.6)	(9.7, 15.0)

HI, hemagglutination-inhibition; CI, confidence interval; GMT, geometric mean titer; HI SCR, seroconversion rate (proportion with pre-vaccination titer <1:10 and a post-vaccination titer ≥1:40, or a pre-vaccination titer ≥1:10 and at least a four-fold increase in post-vaccination titer); SPR, seroprotection rate (as proportion of subjects with HI antibody titers ≥ 1:40); SCF, seroconversion factor (geometric mean of the within subject ratios of reciprocal HI antibody titers for post-vaccination versus pre-vaccination). All participants received inactivated trivalent influenza vaccine, non-adjuvanted (non-adjuvanted ≥65 years and control 18–40 years) or formulated with an adjuvant. AS03 is a squalene and α-tocopherol oil-in-water emulsion-based Adjuvant System, with tocopherol content 11.86 mg (A), 5.93 mg (B), or 2.97 mg (C); MPL is 3-O-desacyl-4'-monophosphoryl lipid A: 25 µg (MPL-25) or 50 µg (MPL-50).
