# Supplementary material for: Selection of an adjuvant for seasonal influenza vaccine in elderly people: modelling immunogenicity from a randomized trial
Source: BMC Infect Dis. 2013 Jul 26;13:348. doi: 10.1186/1471-2334-13-348 (PMC3729430; doi:10.1186/1471-2334-13-348)
Supplement: Additional file 3 — Participants reporting solicited local symptoms during the 7-day post-vaccination period in the total vaccinated cohort. [file 1471-2334-13-348-S3.pdf]

Additional file 3. Participants reporting solicited local symptoms during the 7-day post-vaccination period in the total vaccinated cohort

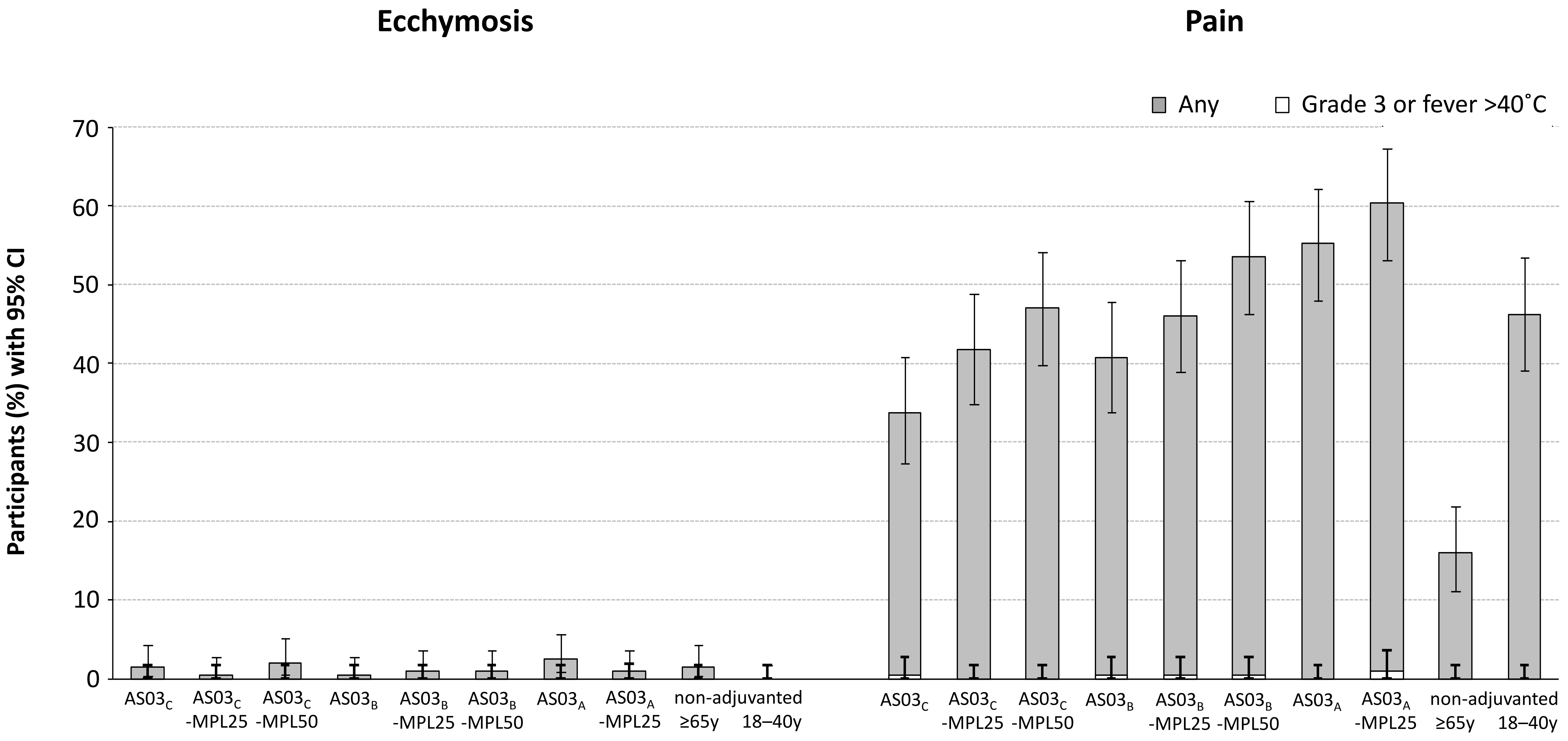

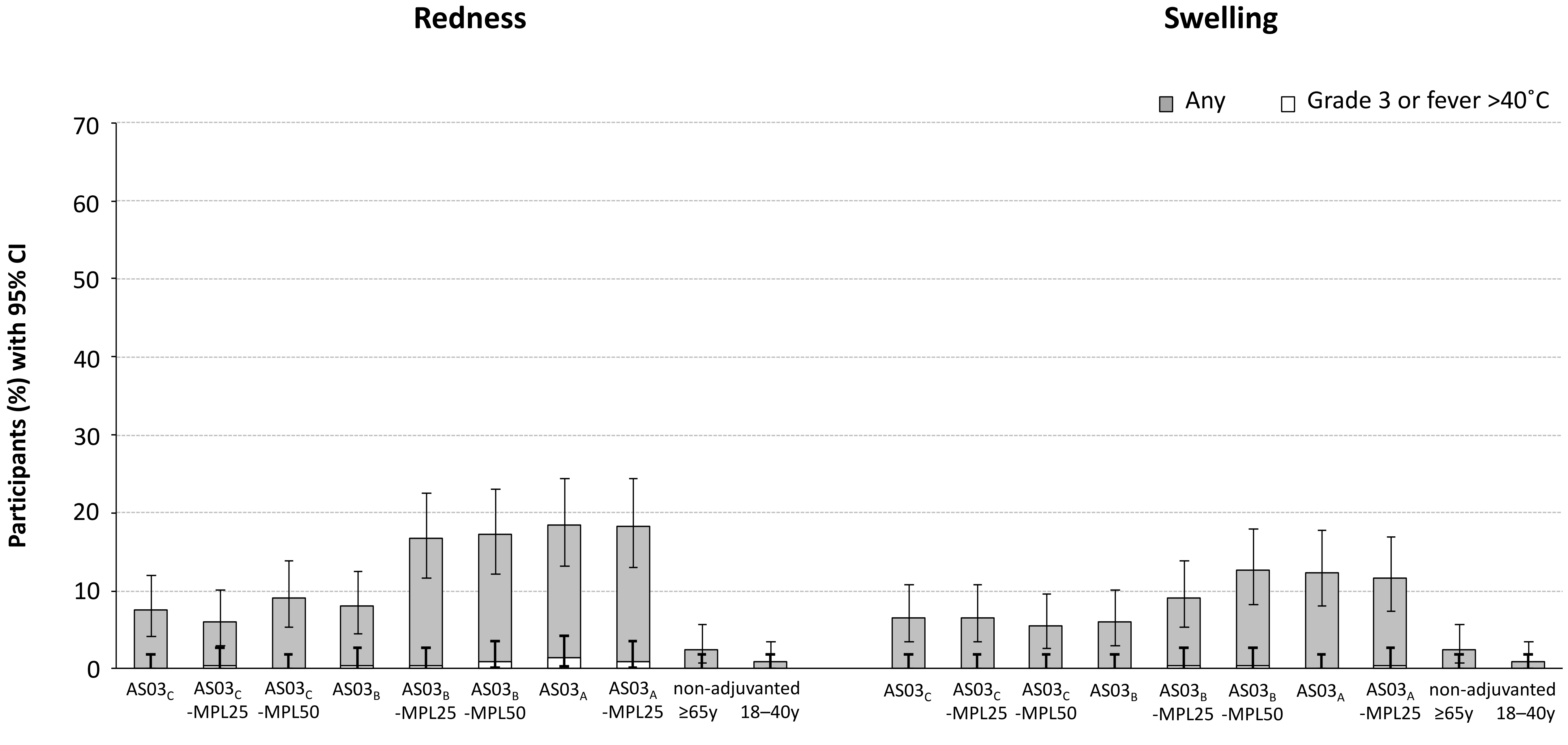

All participants received inactivated trivalent influenza vaccine, non-adjuvanted (non-adjuvanted ≥65 years and control 18–40 years) or formulated with an adjuvant. AS03 is a squalene and α-tocopherol oil-in-water emulsion-based Adjuvant System, with tocopherol content 11.86 mg (A), 5.93 mg (B), or 2.97 mg (C); MPL is 3-O-desacyl-4’- monophosphoryl lipid A: 25 µg (MPL-25) or 50 µg (MPL-50).
